# Supplementary material for: Weak Cross-Lineage Neutralization by Anti SARS-CoV-2 Spike Antibodies after Natural Infection or Vaccination Is Rescued by Repeated Immunological Stimulation
Source: Vaccines (Basel). 2021 Oct 2;9(10):1124. doi: 10.3390/vaccines9101124 (PMC8537215; doi:10.3390/vaccines9101124)
Supplement: Supplementary file 1 [file vaccines-09-01124-s001.zip › vaccines-1357977-supplementary/Supplemental figures.pdf]

# SUPPLEMENTAL FIGURES

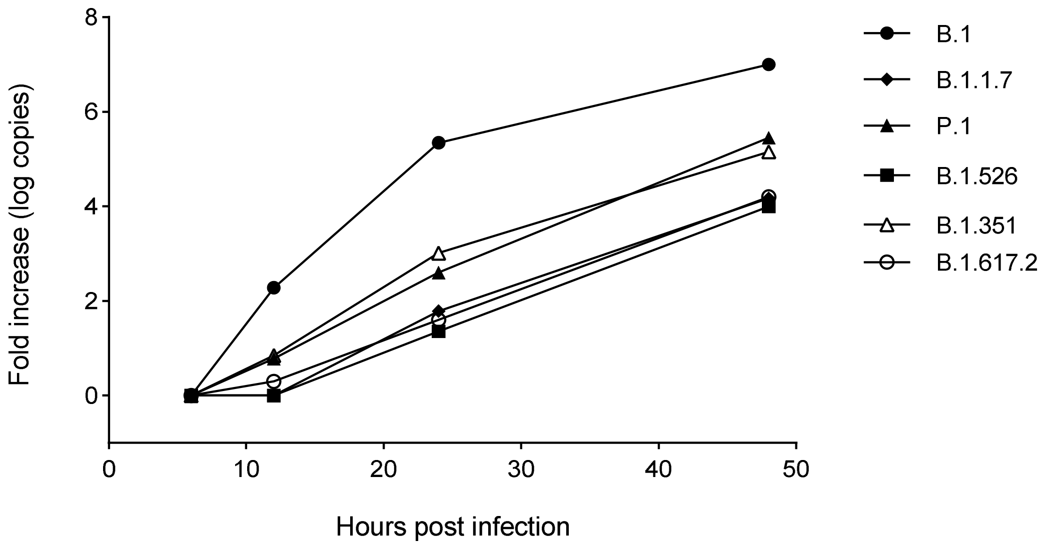


**Supplemental figure S1**: Different lineages of SARS-CoV-2 kinetic growth in Vero E6 cells after 6, 12, 24, 48 hours post infection; Data are expressed as mean values of six replicates.

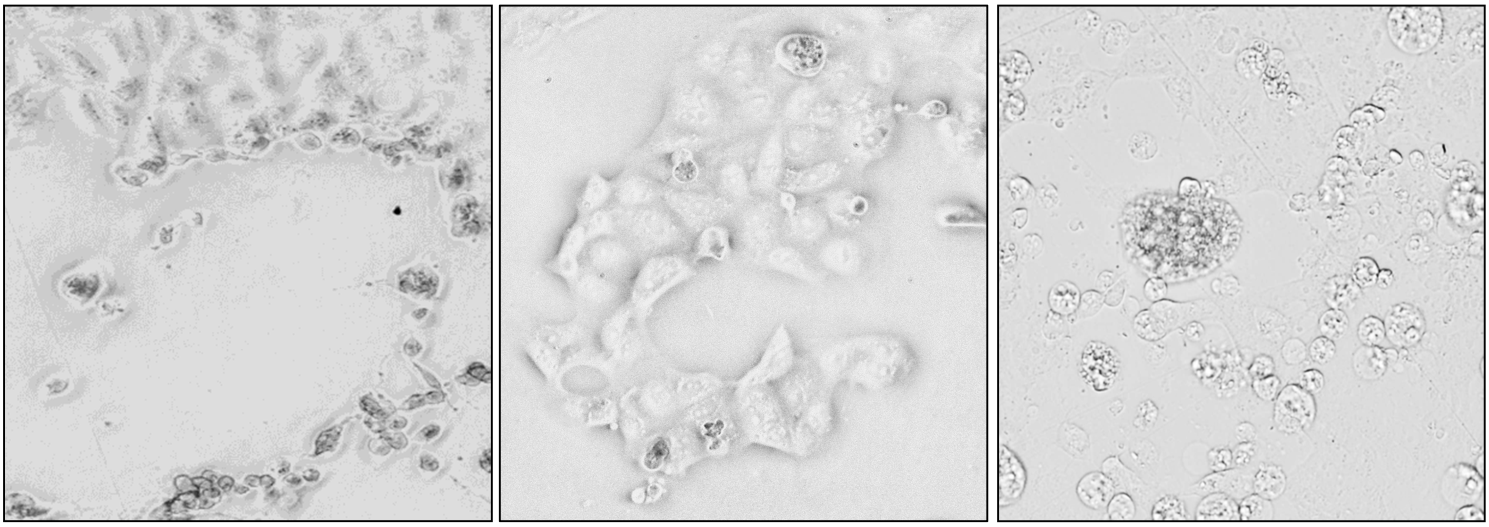

**Supplemental figure S2**: Cytopathic effects (CPE) of SARS-CoV-2 lineages in Vero E6 cells. CPE consisting of rounded-shape, refractile cells undergoing a detachment.

On the left, Vero E6 cells infected with the B.1 lineage; at the centre, a more syncytiogenic CPE was observed after infection of Vero cells with the B.1.1.7 lineage, on the right syncytia were even more evident with the B.1.617.2 lineage, where the typical ballooning of dying syncytia is visible.
