# Supplementary material for: Weak Cross-Lineage Neutralization by Anti SARS-CoV-2 Spike Antibodies after Natural Infection or Vaccination Is Rescued by Repeated Immunological Stimulation
Source: Vaccines (Basel). 2021 Oct 2;9(10):1124. doi: 10.3390/vaccines9101124 (PMC8537215; doi:10.3390/vaccines9101124)
Supplement: Supplementary file 1 [file vaccines-09-01124-s001.zip › vaccines-1357977-supplementary/Supplemental table.pdf]

Serum ID	Date of serum collection	Sex	Age	Date of SARS-CoV-2 infection	Infection with lineages	Dato of vaccionation	Reciprocal plasma dilution values						Anti-nucleocapsid Ig tot	Anti-spike IgG II BAU/ml
							B.1	B.1.1.7	P.1	B.1.526	B.1.351	B.1.617.2		
**kers receiving BNT162b2 vaccine (I dose)**														
30850	02/02/2021	F	33			12/01/2021	453	345	26	<20	<20	<20	Non reactive	374,48
77165	02/02/2021	F	31			12/01/2021	12	<20	12	<20	<20	<20	Non reactive	281,78
34284	04/02/2021	F	60			14/01/2021	29	<20	<20	<20	<20	<20	Non reactive	248,41
77475	02/02/2021	F	24			12/01/2021	113	45	71	<20	<20	<20	Non reactive	221,53
72796	26/01/2021	F	67			05/01/2021	26	<20	<20	<20	<20	<20	Non reactive	178,97
79330	26/01/2021	F	39			05/01/2021	14	<20	<20	<20	<20	<20	Non reactive	167,54
78562	28/01/2021	F	40			07/01/2021	37	<20	<20	<20	<20	<20	Non reactive	126,87
31249	31/01/2021	M	40			10/01/2021	<20	<20	<20	<20	<20	<20	Non reactive	124,8
21380	01/02/2021	F	57			11/01/2021	<20	<20	<20	<20	<20	<20	Non reactive	117,83
11112	28/01/2021	M	56			07/01/2021	12	<20	12	<20	<20	<20	Non reactive	104,66
57847	04/02/2021	F	55	28/02/2020	unknown	14/01/2021	1194	594	384	139	96	139	Reactive	1808,78
32819	03/02/2021	F	32	03/11/2020	unknown	13/01/2021	>1280	689	384	457	113	267	Reactive	2260.69
75818	05/02/2021	F	32	15/10/2020	unknown	15/01/2021	2153	877	594	381	172	206	Reactive	2668.90
**kers receiving BNT162b2 vaccine (II dose)**														
42463	17/02/2021	F	50			13/01/2021	99	57	261	9	<20	11	Non reactive	784,96
34764	11/02/2021	F	54			07/01/2021	113	42	67	36	<20	12	Non reactive	769,27
35037	10/02/2021	F	55			05/01/2021	113	48	172	43	<20	8	Non reactive	863,06
42066	15/02/2021	F	57			11/01/2021	192	74	172	64	<20	21	Non reactive	1960,35
42043	17/02/2021	M	40			10/01/2021	113	34	88	52	<20	26	Non reactive	827,91
36868	11/02/2021	F	40			07/01/2021	533	297	533	82	14	88	Non reactive	3651,43
41772	09/02/2021	F	67			05/01/2021	345	149	345	94	28	33	Non reactive	1318,71
33066	12/02/2021	F	56			05/01/2021	351	149	160	56	<20	71	Non reactive	990,32
41822	18/02/2021	F	33			12/01/2021	640	345	533	80	<20	53	Non reactive	2709,84
40717	09/02/2021	F	39			05/01/2021	987	258	987	452	37	94	Non reactive	3743,53
42384	18/02/2021	F	60			14/01/2021	533	301	604	359	14	67	Non reactive	4397,14
34302	12/02/2021	F	59			09/01/2021	987	297	521	292	43	297	Non reactive	5384,69
38174	22/02/2021	F	46			18/01/2021	226	113	160	79	<20	9	Non reactive	4309,65
57977	18/02/2021	M	31			14/01/2021	172	74	37	111	<20	96	Non reactive	1884,63
33417	19/02/2021	F	52			15/01/2021	384	113	86	144	<20	47	Non reactive	1225,19
57924	18/02/2021	M	63			14/01/2021	172	74	34	33	<20	28	Non reactive	1024,13
33589	19/02/2021	F	61			15/01/2021	226	43	12	43	<20	74	Non reactive	947,23
57482	15/02/2021	M	32			13/01/2021	594	172	67	175	28	44	Non reactive	2154,38
37497	10/03/2021	F	55			13/01/2021	113	26	<20	57	<20	20	Non reactive	574,57
57711	15/02/2021	F	50			12/01/2021	149	12	26	105	<20	10	Non reactive	390,11
57530	16/02/2021	M	51			12/01/2021	149	34	26	40	<20	20	Non reactive	747,18
33382	19/02/2021	F	52			14/01/2021	594	226	172	278	14	82	Non reactive	3722,31
50748	15/02/2021	M	59			10/01/2021	689	538	345	345	48	53	Non reactive	4344,83
59047	02/03/2021	F	45			15/01/2021	905	226	149	94	37	80	Non reactive	2171,59
36864	17/02/2021	F	24			12/01/2021	453	226	267	265	86	26	Non reactive	3128,87
36831	16/02/2021	F	26			12/01/2021	351	113	96	68	<20	26	Non reactive	2703,38
45394	12/02/2021	M	56			07/01/2021	86	57	42	43	<20	26	Non reactive	1647,15
36841	16/02/2021	F	31			12/01/2021	464	297	172	202	57	27	Non reactive	10447,36
76252	15/02/2021	F	61			11/01/2021	297	86	34	58	<20	22	Non reactive	2317,31
33595	19/02/2021	F	54			15/01/2021	453	258	113	110	43	113	Non reactive	2801,34
43451	17/02/2021	F	36			13/01/2021	226	57	86	74	37	12	Non reactive	2823
43889	18/02/2021	F	60			13/01/2021	384	113	86	345	34	48	Non reactive	3273,35
44681	19/02/2021	F	49			15/01/2021	267	43	37	38	<20	25	Non reactive	919,71
43595	18/02/2021	F	43			12/01/2021	345	113	86	87	<20	26	Non reactive	2382,22
43918	18/02/2021	F	44			14/01/2021	453	86	48	227	<20	37	Non reactive	2284,42
44494	19/02/2021	M	49			14/01/2021	192	57	47	48	<20	18	Non reactive	1984
43775	18/02/2021	F	39			13/01/2021	689	172	86	115	<20	113	Non reactive	1938,41
44456	19/02/2021	F	35			13/01/2021	198	43	37	42	<20	49	Non reactive	1207,24
43264	17/02/2021	F	53			13/01/2021	147	17	<20	17	<20	44	Non reactive	636,11
43752	18/02/2021	F	58			12/01/2021	905	351	113	363	43	137	Non reactive	4251,59
50058	18/03/2021	M	56			11/02/2021	594	453	113	179	34	90	Non reactive	4651,32
39751	18/03/2021	F	58			11/02/2021	594	172	86	148	26	44	Non reactive	3998,62
49535	18/03/2021	F	50			11/02/2021	2153	297	172	976	34	269	Non reactive	5483,18
49500	18/03/2021	F	49			10/02/2021	226	43	26	57	<20	26	Non reactive	1901,87
49592	18/03/2021	M	28			11/02/2021	384	172	113	96	37	20	Non reactive	5124,96
50005	18/03/2021	F	42			11/02/2021	761	453	521	290	74	88	Non reactive	5503,89
44351	18/03/2021	M	50			11/02/2021	2153	172	297	353	74	160	Non reactive	5041,56
36631	18/03/2021	F	56			11/02/2021	2153	594	226	707	86	353	Non reactive	5274,05
41757	23/02/2021	M	59			10/01/2021	689	453	297	479	74	227	Non reactive	3618,98
57493	02/03/2021	F	34			16/01/2021	43	12	<20	9	<20	12	Non reactive	628,23
57848	23/02/2021	F	55	28/02/2020	unknown	14/01/2021	>1280	761	689	594	149	689	Reactive	2561,52
42090	19/02/2021	F	32	03/11/2020	unknown	13/01/2021	2153	594	453	190	113	587	Reactive	3143,22
44588	19/02/2021	F	32	15/10/2020	unknown	15/01/2021	>1280	1076	>1280	594	248	689	Reactive	4906,46
49531	18/03/2021	F	57	28/02/2020	unknown	11/02/2021	>1280	2153	2153	453	538	453	Reactive	4778,1
41765	23/02/2021	F	55	27/10/2020	unknown	16/01/2021	>1280	1522	570	594	176	345	Reactive	3638,96
45170	25/02/2021	F	31	28/02/2020	unknown	04/01/2021	2153	1522	453	294	113	453	Reactive	2578,76
69351	05/03/2021	F	40	14/12/2020	unknown	15/01/2021	726	533	297	65	43	76	Reactive	2816,08
36231	10/03/2021	M	59	24/10/2020	unknown	13/01/2021	>1280	987	1522	453	172	594	Reactive	2637,38
45203	25/02/2021	F	25	28/02/2020	unknown	16/01/2021	>1280	2153	987	810	594	761	Reactive	4090,65
39885	23/02/2021	F	42	28/02/2020	unknown	12/01/2021	>1280	594	538	345	113	421	Reactive	2546,1
41741	23/02/2021	F	55	04/04/2020	unknown	16/01/2021	>1280	2153	761	986	226	380	Reactive	2198,4
44042	24/02/2021	F	42	12/11/2020	unknown	08/01/2021	2153	384	229	302	37	149	Reactive	1809,23
44525	24/02/2021	M	32	28/02/2020	unknown	07/01/2021	>1280	905	564	345	393	895	Reactive	2301,8
48548	26/02/2021	M	51	21/10/2020	unknown	12/01/2021	2153	689	384	922	113	250	Reactive	2762,14
45341	25/02/2021	F	48	05/10/2020	unknown	10/01/2021	>1280	>1280	594	223	149	320	Reactive	2327,22
41436	23/02/2021	F	35	09/03/2021	B.1.1.7	05/01/2021	172	113	28	43	12	56	Non reactive	2217,38
37599	22/02/2021	M	61	11/03/2021	B.1.1.7	17/01/2021	90	43	34	34	<20	11	Non reactive	1852,27
37563	22/02/2021	M	24	04/03/2021	B.1.1.7	10/01/2021	149	133	43	44	<20	19	Non reactive	2072,84
59234	02/03/2021	F	50	13/03/2021	B.1.1.7	15/01/2021	172	67	48	43	<20	43	Non reactive	1639,4
55279	01/03/2021	F	50	17/03/2021	B.1.1.7	13/01/2021	172	113	34	57	26	43	Non reactive	1485,88
60133	03/03/2021	F	36	18/03/2021	B.1.1.7	10/01/2021	384	113	37	36	12	106	Non reactive	1910,58
74994	12/03/2021	F	24	16/02/2021	B.1.1.7	04/01/2021	453	133	99	176	34	63	Non reactive	1496,33
44097	24/02/2021	M	39	01/05/2021	B.1.1.7	04/01/2021	198	192	176	40	18	28	Non reactive	2304,2
74815	15/04/2021	M	79	04/06/2021	B.1.351	05/03/2021	453	297	987	297	297	113	Non reactive	1109,87
55464	04/03/2021	F	36	07/07/2021	B.1.617.2	07/01/2021	698	351	987	594	86	297	Non reactive	4386,6
40498	23/02/2021	F	55	05/07/2021	B.1.1.7	15/01/2021	172	74	226	57	<20	34	Non reactive	1873,2
57340	20/04/2021	F	31	08/07/2021	unknown	22/03/2021	640	160	80	226	80	113	Non reactive	1607,26
**taff receiving AZD1222 vaccine (I dose)**														
29353	14/04/2021	M	38			24/03/2021	14	<20	<20	<20	<20	<20	Non reactive	222,95
29366	14/04/2021	M	68			24/03/2021	12	<20	<20	<20	<20	<20	Non reactive	100,33
29354	14/04/2021	F	62			24/03/2021	26	<20	<20	<20	<20	<20	Non reactive	97,24
29378	14/04/2021	M	65			15/03/2021	26	<20	<20	<20	<20	<20	Non reactive	70,48
29339	14/04/2021	F	33			15/03/2021	12	<20	<20	<20	<20	<20	Non reactive	60,9
29312	14/04/2021	F	39			15/03/2021	12	<20	<20	<20	<20	<20	Non reactive	46,9
29372	14/04/2021	M	61			15/03/2021	<20	<20	<20	<20	<20	<20	Non reactive	37,41
29325	14/04/2021	M	59			15/03/2021	14	<20	<20	<20	<20	<20	Non reactive	34,81
29308	14/04/2021	F	43			15/03/2021	26	<20	<20	<20	<20	<20	Non reactive	26,21
29343	14/04/2021	F	34			24/03/2021	<20	<20	<20	<20	<20	<20	Non reactive	23,28
27104	02/04/2021	F	48	01/03/2020	unknown	08/03/2021	2153	905	297	579	453	579	Reactive	1116,71
26434	31/03/2021	M	26	16/03/2020	unknown	10/03/2021	2153	698	761	297	133	226	Reactive	1546,57
26457	31/03/2021	M	45	23/10/2020	unknown	10/03/2021	2153	594	564	113	57	72	Reactive	3119,04
26490	31/03/2021	F	52	16/03/2020	unknown	10/03/2021	>1280	689	640	345	113	205	Reactive	1793,11
26492	31/03/2021	M	60	16/03/2020	unknown	10/03/2021	>1280	2153	>1280	>1280	987	2153	Reactive	8469,03
26767	01/04/2021	F	46	30/10/2020	unknown	08/03/2021	226	74	96	14	<20	22	Reactive	491,6
27081	02/04/2021	F	59	29/10/2020	unknown	12/03/2021	>1280	453	905	761	297	453	Reactive	2162,44
27102	02/04/2021	F	33	11/11/2020	unknown	12/03/2021	>1280	726	905	987	345	689	Reactive	2344,63
29332	14/04/2021	M	42	16/03/2020	unknown	15/03/2021	>1280	2153	2153	905	359	689	Reactive	3042,91
29694	15/04/2021	M	59	07/12/2020	unknown	25/03/2021	533	297	172	192	74	67	Reactive	1043,88
**taff receiving AZD1222 vaccine (II dose)**														
24960	21/06/2021	F	33			15/03/2021	113	43	43	43	12	9	Non reactive	175,21
24950	21/06/2021	F	39			15/03/2021	74	43	57	57	26	15	Non reactive	186,8
24980	21/06/2021	M	61			15/03/2021	192	74	113	43	<20	13	Non reactive	618,49
24963	21/06/2021	M	59			15/03/2021	86	57	113	74	<20	16	Non reactive	317,54
24956	21/06/2021	F	43			15/03/2021	67	34	86	37	12	17	Non reactive	139,89
23282	14/06/2021	M	26	16/03/2020	unknown	10/03/2021	2153	594	533	143	149	250	Reactive	1033,27
22380	10/06/2021	F	52	16/03/2020	unknown	10/03/2021	689	453	345	226	86	46	Reactive	1331,42
22333	10/06/2021	F	46	30/10/2020	unknown	08/03/2021	226	74	86	26	<20	8	Reactive	429,02
**Convalescent sera**														
89870	19/06/2020	M	59	29/03/2020	B.1		226	57	57		37		Reactive	1007,24
85257	25/06/2020	M	54	13/03/2020	B.1		37	24	14		<20		Reactive	416,70
85253	25/06/2020	M	56	30/03/2020	B.1		113	43	62		<20		Reactive	781,09
85269	25/06/2020	M	36	16/03/2020	B.1		173	28	57		<20		Reactive	466,60
85556	29/06/2020	M	46	25/03/2020	B.1		226	86	34		26		Reactive	349,13
85309	25/06/2020	M	57	28/03/2020	B.1		226	62	48		26		Reactive	452,85
85945	03/07/2020	M	40	20/04/2020	B.1		45	<20	<20		<20		Reactive	107,49
85981	04/07/2020	M	57	10/04/2020	B.1		43	<20	<20		<20		Reactive	24,90
86042	04/07/2020	M	52	09/04/2020	B.1		57	28	17		<20		Reactive	270,91
86152	06/07/2020	M	56	18/03/2020	B.1		226	48	113		12		Reactive	432,21
86162	07/07/2020	M	48	21/03/2020	B.1		295	57	48		<20		Reactive	409,23
86250	07/07/2020	M	31	28/03/2020	B.1		90	28	<20		<20		Reactive	179,01
86202	07/07/2020	M	47	27/03/2020	B.1		146	28	<20		<20		Reactive	140,37
86565	11/07/2020	M	54	30/03/2020	B.1		267	113	74		14		Reactive	106,00
86596	13/07/2020	F	34	10/03/2020	B.1		240	37	48		<20		Reactive	264,47
86766	14/07/2020	M	40	13/05/2020	B.1		43	34	12		<20		Reactive	320,40
86997	17/07/2020	M	62	28/03/2020	B.1		226	57	74		<20		Reactive	287,53
87104	18/07/2020	M	34	05/04/2020	B.1		65	34	12		<20		Reactive	460,46
87152	20/07/2020	M	60	15/04/2020	B.1		99	28	12		<20		Reactive	428,60
87644	24/07/2020	M	32	24/03/2020	B.1		295	37	37		<20		Reactive	187,27
87703	27/07/2020	M	53	19/03/2020	B.1		226	37	28		<20		Reactive	460,24
87959	29/07/2020	M	51	07/03/2020	B.1		93	12	35		<20		Reactive	235,79
88179	31/07/2020	M	37	10/03/2020	B.1		113	37	12		<20		Reactive	256,84
88207	01/08/2020	M	53	24/03/2020	B.1		453	86	149		28		Reactive	200,04
88478	05/08/2020	F	36	24/03/2020	B.1		192	48	<20		<20		Reactive	124,70
88777	08/08/2020	M	38	15/03/2020	B.1		297	37	113		12		Reactive	458,30
89195	17/08/2020	M	53	15/03/2020	B.1		226	48	74		<20		Reactive	186,77
89419	19/08/2020	M	56	22/03/2020	B.1		226	57	43		<20		Reactive	199,00
89675	22/08/2020	M	56	16/04/2020	B.1		207	149	37		<20		Reactive	1050,90
85171	29/08/2020	M	47	27/03/2020	B.1		149	37	43		14		Reactive	172,91
85632	04/09/2020	F	34	17/03/2020	B.1		74	48	12		<20		Reactive	269,36
85805	07/09/2020	M	56	29/03/2020	B.1		149	43	26		<20		Reactive	317,90
85886	08/09/2020	M	58	05/03/2020	B.1		226	74	74		26		Reactive	169,74
74707	12/03/2021	M	47	28/01/2021	B.1.1.7		86	172	26	28	26	26	Reactive	200,55
73952	11/03/2021	M	23	08/02/2021	B.1.1.7		149	226	113	38	45	13	Reactive	917,18
69243	06/03/2021	M	49	18/02/2021	B.1.1.7		267	905	172	44	26	52	Reactive	2394,24
33511	03/04/2021	M	44	19/02/2021	B.1.1.7		113	285	143	34	14	57	Reactive	658,91
40333	27/03/2021	M	51	01/03/2021	B.1.1.7		594	761	226	389	96	191	Reactive	1033,01
58371	01/04/2021	M	69	18/03/2021	B.1.1.7		415	291	57	89	45	136	Reactive	1311,41
47066	26/03/2021	F	41	08/02/2021	B.1.1.7		226	297	48	226	12	57	Reactive	1515,17
56234	01/04/2021	F	68	10/03/2021	B.1.1.7		96	359	34	48	17	15	Reactive	2482,11
69618	02/04/2021	M	75	10/02/2021	B.1.1.7		761	987	226	500	113	771	Reactive	1517,5
57052	31/03/2021	F	67	18/03/2021	B.1.1.7		192	226	74	37	57	73	Reactive	2086,27
26829	02/04/2021	M	34	02/03/2021	B.1.1.7		57	400	57	31	<20	17	Reactive	418,22
42151	25/03/2021	M	54	04/02/2021	B.1.1.7		453	396	64	385	43	345	Reactive	1361,47
69952	02/04/2021	M	67	27/02/2021	B.1.1.7		113	57	28	14	<20	16	Reactive	636,1
45881	14/04/2021	M	54	07/03/2021	P.1		479	226	877	439	133	110	Reactive	814,52
43206	21/04/2021	F	47	11/02/2021	B.1.351		86	57	62	43	74	6	Reactive	135,6
